# Integrated community case management of malaria, pneumonia and diarrhoea across three African countries: A qualitative study exploring lessons learnt and implications for further scale up

**DOI:** 10.7189/jogh.04.020404

**Published:** 2014-12

**Authors:** Clare Strachan, Alexandra Wharton–Smith, Chomba Sinyangwe, Denis Mubiru, James Ssekitooleko, Joslyn Meier, Miatta Gbanya, James K. Tibenderana, Helen Counihan

**Affiliations:** 1 Malaria Consortium Africa Regional Office, Kampala, Uganda; 2 Malaria Consortium Zambia, Mansa, Luapula Province, Zambia; 3 Malaria Consortium Uganda, Kampala, Uganda; 4 Malaria Consortium South Sudan, Juba, South Sudan

## Abstract

Numerous studies highlight the effectiveness of an integrated approach for the management of malaria, pneumonia and diarrhoea at the community level. There has however been little study on lessons learnt from implementation in practice and stakeholder experiences which could inform future programmatic planning and evaluation frameworks. A participatory, qualitative evaluation was conducted in the three varied settings of South Sudan, Uganda and Zambia, which have seen the scale up of integrated community case management (iCCM) over the last five years. All key in–country stakeholders were consulted on study design, with a particular focus on scope and methodology. Data collection methods included stakeholder consultations (key informant interviews, focus group discussions), and a review of project and Ministry of Health documentation. Data analysis followed the Framework Approach. Results suggest that iCCM implementation generally followed national pre–agreed guidelines. Overarching key programmatic recommendations included: collaboration with implementing partners in planning stages to positively impact on community acceptance and ownership; adoption of participatory training methods adapted to low literacy populations; development of alternative support supervision methods such as peer support groups; full integration of community level data into the health management information system and emphasizing data analysis, use and feedback at all levels; strengthened supply chains through improved quantification and procurement of commodities in conjunction with the national distribution network; community engagement to establish a support system for community health workers to increase their motivation; enhanced sensitisation and behaviour change communication to raise awareness and usage of appropriate health services; and advocacy at the national level for funding and logistical support for the continuation and integration of iCCM. This qualitative study is a valuable contribution in understanding the ‘hows’ of iCCM implementation with key insights for improved feasibility and acceptability. Main findings show how community support to iCCM and community health workers is necessary for sustained health benefits coupled with a focus on strengthening and ‘enabling’ the public health system. The participatory study design and methodologies used enabled the scope of the research enquiry to effectively capture various stakeholder perspectives.

Integrated community case management (iCCM) delivered through trained community health workers (CHWs) can contribute to the reduction in morbidity and mortality of the three major causes of mortality for children globally under the age of five, specifically pneumonia (18%), diarrhoea (15%) and malaria (8%) [1]. iCCM typically provides free community level treatment for these diseases to children aged two months to five years. In many remote and hard–to–reach areas, this can improve health outcomes, providing a potentially faster clinical response after onset of signs and symptoms. It also saves on caregiver time and transportation costs when seeking medical care [2-4]. Simple diagnostic tools, including rapid diagnostic tests (RDTs) for malaria and respiratory rate counters for pneumonia, can be used by CHWs to identify illnesses which are then treated with artemisinin–based combination therapy, oral antibiotics or oral rehydration therapy and zinc, dependent upon the patient history and test results.

iCCM has been a growing focus of community health care delivery across Sub–Saharan Africa over the last few years and gained momentum in the wake of an increasing body of research [2], which has highlighted the effectiveness of an integrated approach at the community level in reducing under–five morbidity and mortality caused by malaria, pneumonia and diarrhoeal diseases.

Evaluation of iCCM in its formative years has focused on important quantitative outcomes and impact [5], specifically morbidity, mortality and lives saved [6]. Separate work has also been conducted on the financing of iCCM and the implications for larger scale implementation [7]. Qualitative work has tended to explore community acceptability [8], CHW motivation and effectiveness, in addition to the role of community based and technological innovations in improving service delivery [9-11]. It is recognised that more research is needed to determine the best approaches to iCCM implementation, scale up and sustainability and to capture beneficiary experiences [12]. The aim of this participatory evaluation was to capture context specific experiences of a range of stakeholders in South Sudan, Uganda and Zambia, where Malaria Consortium has supported the Ministry of Health (MOH) to introduce and implement iCCM over the past three years.

## METHODS

The study adopted a qualitative approach to enable an in–depth exploration of a range of stakeholder experiences of iCCM implementation, utilising a participatory approach as far as possible. Data was collected through stakeholder consultations and beneficiary assessments, specifically key informant interviews (KIIs) and focus group discussions (FGDs), as well as an analysis of Malaria Consortium and MOH documentation. Data collection was conducted from November 2012 to March 2013 in South Sudan, Uganda and Zambia. In Uganda, the evaluation was seen as a key contribution to the national iCCM review led by the MOH with main stakeholders.

### Study setting

Since 2010, iCCM has been implemented by Malaria Consortium, according to MOH guidelines, in collaboration with the MOH in the Central and Western regions of Uganda, in the northern Luapula province in Zambia, and in Unity and Northern Bahr el Ghazal states in South Sudan. The majority of beneficiaries across implementation sites reside in rural areas, with very limited access to the formal health service.

### Scope of enquiry and participation selection

Thematic areas of enquiry to guide and structure the data collection process were developed. These were based on the established phases of iCCM start up and implementation (as indicated in most national iCCM guidelines where available), as well as additional thematic categories relating to the relevance of iCCM, namely acceptability, effectiveness and sustainability. Within each theme, specific enquiry explored the implementation process, aspects which worked well, challenges faced and recommendations in relation to continued/future iCCM implementation. The approach used for this was participatory evaluation which is a partnership approach to evaluation where stakeholders actively engage in developing the evaluation and phases of its implementation [13]. As part of the consultative nature of the participatory evaluation, key in–country implementers, specifically MOH staff active at different levels of the health system, CHWs, implementing partners and beneficiaries, were engaged through workshops to discuss and agree the thematic scope relevant to context, data collection methods and specific interview targets of the evaluation. Specific topic guides were developed and piloted with each identified target group.

A maximum variation sampling approach [14] was followed to ensure a sufficient range of respondents were included according to factors which are likely to represent a diversity of views. The participants were categorised into target groups based on their role in iCCM implementation as outlined in **Table 1**. In total, 646 participants were included in the evaluation, (Zambia: 241, Uganda: 217, South Sudan: 188).

**Table 1:** Selection criteria for study inclusion by target group

Target Group	Selection criteria
**Malaria Consortium staff**	Played a key role in iCCM implementation
**MOH central level**	Supported iCCM planning, implementation
**MOH sub–national level (province/state/district/payam)**	Supported iCCM planning, implementation
**District Health Management Team (DHMT)**	District Medical Officers, Pharmacists, DHMT members involved in iCCM
**Health facility staff**	Clinical staff who are involved in managing referred outpatient and inpatient cases of malaria, pneumonia and diarrhoea and were available on the day of interview from one central and one rural clinic in each district
**Community leaders**	Village chiefs, headmen, church elders, village committee chairmen, who have been involved in iCCM implementation. In each district, one FGD of purposively selected community leaders from rural and urban areas
**Community health workers**	iCCM trained CHWs. In each district: one FGD with CHWs attached to each of the two selected health facilities
**Beneficiaries**	Caregivers of children under the age of five. In each district two FGDs of beneficiaries residing in the catchment areas of the selected health facilities were randomly selected

iCCM – integrated community case management, MOH – Ministry of Health, CHW – community health worker, FGD – focus group discussion

Malaria Consortium staff, MOH officials at each level, District Health Management Teams (DHMT) (Zambia), District Health Teams (DHT) (Uganda) and health facility staff were purposively selected in relation to their involvement in iCCM planning and implementation. In Luapula province, Zambia, Mansa, Kawambwa, Chienge and Samfya districts were sampled; in Uganda, Hoima, Buliisa and Mpigi districts were included; in South Sudan, Aweil West and Aweil Centre counties in Northern Bahr el Ghazal state were sampled. Villages were stratified by distance to health facility (more than five kilometres, less than five kilometres) and their location (hard to reach, rural and peri–urban). In villages where household lists were unavailable, households with caregivers of children under the age of five were chosen by the random walk technique. CHWs, their supervisors and community leaders residing in the selected villages were invited to the FGDs. The number of interviews, FGDs and observations were estimated based on what is required in order to reach data saturation, balanced with the time and resources available.

### Data collection

KIIs were conducted with central and provincial level MOH officials and with Malaria Consortium staff in all three countries, and for community leaders and health facility in–charges (in Uganda), to explore implementation experiences in more depth.

Separate FGDs were considered preferable for District Health Management Teams/District Health Teams, payam administrators (South Sudan), health facility staff, CHWs and their supervisors, community leaders (in South Sudan and Zambia) and beneficiaries to stimulate discussion in groups that would not feel inhibited by power hierarchies.

A total of 72, 20 and 64 CHWs were sampled from South Sudan, Uganda and Zambia respectively. The number of beneficiaries sampled in South Sudan was 72, in Uganda 81 and in Zambia it was 145. In South Sudan, a total of 23 FGDs and 7 KIIs were held, in Uganda there were 36 FGDs and 27 KIIs while in Zambia the numbers were 26 FGDs and 25 KIIs.

The data collectors/transcribers were recruited using criteria which stipulated previous experience in qualitative research data collection. All members of the research team participated in a 2-day training on ethics, data collection methods, transcription and data management.

### Grey literature review

MOH data, guidelines, policies and Malaria Consortium iCCM documents such as baseline survey results, training materials, implementation tools, programme assessments, progress and project reports and evaluations were reviewed to provide insight into the implementation process and to add context to experiences of iCCM planning, implementation and evaluation in practice. Evaluations and documented lessons learned from other iCCM implementing partners were also reviewed. Review of grey literature contributed to informing on the background and context for the research team. Relevant content from selected documentation was analysed and contrasted with accounts from respondents, notably on iCCM implementation processes compared to the national guidelines.

### Data management and analysis

All interviews and FGDs were audio recorded and transcribed verbatim by the researchers in the field as soon as possible after the data had been collected for the purpose of maintaining data validity. The transcription process was quality controlled through field review of all transcripts and additional reviews of purposively selected samples by the wider research team. The transcripts were translated into English (where necessary) by the researchers and quality controlled through translation reviews of sections selected at random. Continuous feedback was provided to the researchers throughout the data collection process to support performance improvement. The data were only accessible by the research team and once stored electronically, were anonymised and saved in a password–protected folder shared only with key members of the research team to ensure confidentiality.

The Framework Approach [15] was broadly used to analyse the data. This systematic method appreciates the iterative nature of qualitative data analysis and involves deriving themes related to the research objectives, whilst adding new themes that emerge during data collection, within which the data are analysed and organised. Transcripts were reviewed daily or weekly so as to enable revision during the data collection phase of the scope and questions in the topic guides according to different target groups.

The first level of analysis was conducted by an investigator who reviewed and analysed all of the documentation and transcripts in full, with regular additional analysis by a second investigator. The second investigator compared coding decisions and regularly discussed the scope of data, analysis approach and presentation of the data with the first investigator, with further input from a third senior advisor, as required. When discrepancies arose, the investigators reached a consensus on the most appropriate way to code a passage of text or present the data.

The analysis followed four key stages: identification of key themes during a thorough review of the transcripts, construction of a thematic framework in an Excel spreadsheet for each geographically distinct set of data, which was then used to label and group the data in rows according to themes and sub–themes, emergent subthemes were added to the framework under the relevant overarching themes and the data were once again reviewed and re–sorted, finally, each thematic area was compared between target groups and contextualised, associations between themes were identified; the findings were explained and interpreted. The themes were selected as relevant for each target group. The thematic areas of enquiry and the related subthemes are listed in **Table 2**.

**Table 2:** Thematic areas of enquiry

Thematic area	Subtheme
**Central level preparation**	• Policy and guidelines development process • Introduction of programme to different levels of health system • Other preparation for implementation
**Province/state/district level introduction and start up**	• Steps of introduction and start up • Issues and proposed solutions • Recommendations
**CHW selection**	• Process for recruitment of CHWs for iCCM (agreed approach vs practice) • Appropriateness of selection criteria • Issues which arose, proposed solutions, recommendations
**Training and capacity building**	• Training models including levels of training ie, cascade • Training approaches ie, practical vs theoretical • Training tools • Scope of training in terms of technical content and related learning capacity of CHWs • Evaluation of training • Refresher training • Issues which arose, proposed solutions, recommendations
**Support supervision**	• Models developed, application at different levels • Support supervision tools used • Effectiveness • Alternative supervision models • Issues which arose, proposed solutions, recommendations
**Routine data collection**	• Routine data available, scope and gaps • How data has been collected • How data has been cleaned, stored, summarised, analysed • How data has been used • Extent and scope of feedback given to those involved in submitting and compiling data (including feedback system) • Data quality • Issues which arose, proposed solutions, recommendations
**Commodities and supply chain**	• Push vs pull system • Transport and storage • Packaging • Modes of distribution • Acceptability • Issues which arose, proposed solutions, recommendations
**Community involvement and support**	• Initial community response and changes over time • Community support for CHWs • Role of CHWs and workload/time spent/ motivation, attrition • Utilisation of community iCCM services • Acceptability aspects • Referral uptake • Issues which arose, proposed solutions, recommendations
**Behaviour change communication (BCC)**	• BCC strategy, including scope of BCC activities implemented and why • Perceived/measured value/outcome and impact of activities • Issues which arose, proposed solutions, recommendations
**Management and coordination**	• Project management and staffing structures • Coordination with other projects • Coordination and sharing at the central level and with MOH • Involvement of MOH • Issues which arose, proposed solutions, recommendations
**Integration with health system**	• How the iCCM programme has been integrated • Management of severe cases at health facility level • Support for iCCM among health facility staff • Other aspects relating to sustainability
**Technical and geographical scope of iCCM**	• Scope of services offered and perception of how well this integration works • Appropriate number of CHWs per village • Scale up • Issues which arose, proposed solutions, recommendations
**Evaluation**	• Evaluation methods, process, value • Pilot studies • Other recommendations
**Other**	• Any other feedback

CHW – community health worker, iCCM – integrated community case management, MOH – Ministry of Health

### Ethical considerations

Care was taken by the research team to communicate information about the evaluation to the communities as they worked, in the language and manner that is understandable to them. Informed consent was granted from all participants.

## RESULTS

The results are presented according to key themes related to iCCM implementation components with an emphasis on findings that have implications for improving feasibility, effectiveness and acceptability for future implementation planning and scale up.

In this paper, the generic term of Community Health Worker (CHW) will be used in reference to the Village Health Teams, Community Drug Distributors and Community Health Workers deployed in South Sudan, Uganda and Zambia respectively.

### Central level preparation

At the time of writing, only Uganda had an official national iCCM policy and guidelines, which were drafted by the MOH in collaboration with key implementing partners prior to launching the programme in mid–2010. In Zambia and South Sudan, iCCM currently falls under the wider Integrated Management of Childhood Illness (IMCI) and Child Survival programmes respectively, however an agreed approach for implementation was defined between the MOH and Malaria Consortium. It is understood that developing a formal iCCM policy remains a priority in South Sudan and Zambia. During the preparation stage, specific iCCM training materials, CHW job aids, support supervision tools and data collection forms were developed to facilitate CHW comprehension and promote quality assurance.

Drug resistance due to potential incorrect prescription of medications and poor adherence at community level were highlighted as concerns from stakeholders during the central level planning stages in South Sudan and Zambia. These fears were assuaged by demonstrating how this issue had been successfully dealt with in other iCCM implementation countries through appropriate training of CHWs and effective support supervision. Dialogue meetings with senior MOH and World Health Organisation officials were also considered instrumental in winning support for iCCM and securing stakeholders’ ‘buy in’.

### Sub–national level introduction

The importance of formal introduction from central level MOH at sub–national level was highlighted as key to acceptance among provincial, district and state level officials, particularly where the implementing partner may have not previously worked with local health system stakeholders. Sensitising and mobilising partners at all levels of health governance and service delivery on the iCCM concept, guidelines and processes was identified as integral to the initial phase of implementation and led to higher levels of support at health facilities. Collaboration in micro–planning with lower levels of the MOH including regular participatory sessions to strategise how to address challenges related to training, support supervision, supplies and CHW motivation was greatly valued and was felt to encourage sub national level ownership of the programme. Establishing an agreed framework for ongoing collaboration between district level MOH and implementing partners was considered beneficial to successful coordination.

*“The involvement of district leaders, it was very important … if any programme comes, it has to be owned by the district and therefore sustained … look at ownership and sustainability … if I am the one at the district to go and train those health workers, they don’t look at the programme as from outside … therefore they look at it as theirs and work well on it.”* (District health official, Uganda)

*“Involving all stakeholders including local politicians was very good. Working in partnership with the MoH helped to successfully implement the programme.”* (Malaria Consortium staff)

Initial strengthening of local health team capacity to plan the integration of iCCM activities into budgets and workplans and collect, analyse and utilise iCCM routine data was recommended. Additionally, planning the sustainability of iCCM from the outset was encouraged to adequately prepare for handover to the MOH.

### CHW recruitment and selection

The Ugandan iCCM guidelines and CHW selection documentation from Zambia and South Sudan stipulate that communities should be sensitized on the participatory nature of the recruitment process and selection criteria prior to recruitment. Sensitisation was reported by the majority of respondents as occurring through a variety of channels, most frequently during village meetings, church gatherings, through radio broadcasts and health centre community outreach. Messages emphasized community participation as central to the CHW selection process and listed selection criteria for CHWs as: willingness to volunteer, permanent residency, literacy skills (with the exception of South Sudan), fluency in English (where possible) and being of a reliable and trustworthy disposition. Moreover, that proposed CHWs should not be political or community leaders or their relatives.

Selection of CHWs by communities was preceded by sensitisation meetings with health facility staff and local leaders who explained the selection criteria to communities. Depending on the country context, the majority of community leaders and/or health facility staff facilitated a participatory democratic selection process by presenting interested candidates to the community who were then voted on based on the aforementioned criteria and additional characteristics defined by community members – eg, willingness to volunteer, reliability etc. Sometimes the candidates were selected jointly by the community and leaders (eg, in South Sudan) and in Zambia, priority was given to CHWs previously trained by the MoH.

In all three countries, guidelines mandate that community members should play an active role in choosing their CHWs and not have CHWs imposed by local leaders. Generally, the former participatory approach was followed according to most respondents across countries. In areas where communities were strongly involved in the selection, respondents anecdotally reported higher utilisation of iCCM services, more community support for CHWs, deeper trust in CHWs’ capacity to treat children and a stronger overall sense of community ownership.

“*We were happy because we did the selection as a community and no one imposed them on us … it is good for the people to do the selection because they select someone they trust which is good and if you show someone that you trust him he can do the work well.”* (Community leader, Uganda)

*“... the most important thing in the selection of Community Health Workers is openness. If the people are involved and they feel part of the process, then there will be no problems in the selection process.”* (Community leader, Zambia)

Less democratic approaches occurred in a few communities across all countries, where local leaders would appoint themselves, their relatives or preferred candidate in the interest of personal gain, influence associated with the position, or based on tribal or political affiliations. Tribal dynamics affecting CHW selection was a particular issue in Uganda, where biased selection was reported more frequently than in the other countries. The repercussions of undemocratic selection resulted in unqualified CHWs being sent for training, reluctance among caregivers to access CHWs they felt had been “imposed” upon them or who belonged to a rival political party or tribe and overall less community support for CHWs.

*“The VHT selection was not done on the proper set guidelines... Selection was based on tribal dynamics not on credit. The village members were not involved. The chairpersons took it upon themselves and selected their own people.”* (Health facility staff, Uganda)

Recommendations to encourage community selection included enhanced community sensitisation, consultations with community leaders to discuss the importance of adhering to selection criteria and selection guidelines and selection monitoring by district officials. In Zambia, candidates were required to pass a literacy test and verify their age prior to the training, where district officials played an important role in enforcing democratic selection of appropriate candidates by refusing any unqualified candidate and sending them back to the community for a replacement.

*“We put a system in place and we hope that it works … You have the interesting cases where you have some very old people who are quite influential who will say ‘I will be the Community Health Worker whether people like it or not!’ Nobody would stand up to them and so they would end up coming for the training …”* (Malaria Consortium staff, Zambia)

### Training and capacity building

A cascade training model for iCCM was followed in each country, with a master trainer present for each session for quality assurance. Training duration was for 6 days in all countries and the trainers were health facility and district MoH staff who had attended a Training of Trainers course conducted by central MoH trainers with Malaria Consortium staff. Each training course of CHWs had central MoH or Malaria Consortium staff in attendance for quality assurance. Participatory methods were widely appreciated by trainees and considered conducive to CHW comprehension and subsequent confidence in case management. “Hands on” and practical approaches including group work and discussions, role–plays, and practice using RDTs and actual CHW registers enabled CHWs to gain a solid understanding of the justification behind and practicalities of testing and treatment, according to many respondents. Video sessions were praised as an effective teaching method which gave CHWs the opportunity to observe danger signs without having to wait at a health facility, particularly as some signs, such as “chest indrawing” are difficult to describe verbally or pictorially. In Uganda, the use of dolls for practising the administration of rectal artesunate was valued by CHWs. Visits to health facilities to witness the danger signs associated with severe malaria, pneumonia and diarrhoea cases also strengthened the CHWs’ capacity to recognise these symptoms.

*“I do not know how to write and read, but I have understood the content of the training.”* (CHW, South Sudan)

*“The practical bit of it helps us learn how to handle patients when they visit us. The way you receive someone matters a lot. You might be a health worker but the way you handle me might send me away and I never return.”* (CHW, Uganda)

CHWs appeared to appreciate that facilitators were able to communicate at an appropriate pace, clarifying aspects which were not well understood, and in local languages, which enhanced their understanding of the content. Health facility staff and CHW supervisors remarked on the benefits of supervisory skills training in addition to the iCCM case management they received. The sick child job aid provided to CHWs during the training was referred to as “the Bible” by CHWs in Uganda and Zambia due to its accessibility and comprehensive content.

*“… in relation to first training us on basic knowledge, it was not just a matter of testing but knowing how to deal with VHTs, know how to communicate, technical know–how, … supervise them, and train them and documentation as a result the programme went on smoothly.”* (Health facility staff, Uganda)

Counting the respiratory rate/identification of pneumonia vs cough and data collection using the CHW registers were topics that some CHWs struggled with during the training, according to trainers and CHWs across the countries. Low literacy was commonly mentioned as a challenge for CHWs in South Sudan, however pictorial training materials and CHW registers developed especially for this context facilitated learning and comprehension.

The majority of respondents strongly recommended that the duration of the training be extended to two weeks in order to address some of the more complex topics in more depth, specifically identifying pneumonia, completing CHW registers, correct referral and newborn care. The addition of basic literacy and numeracy skills to the training content was requested by multiple respondents in South Sudan.

Most CHWs and health workers articulated the need for refresher training to consolidate their skills and address any challenges. Several health facility and district level supervisors across the countries indicated that refresher training is motivational for the CHWs. The suggested frequency for refresher training varied widely from monthly to bi–annually.

*“They should bring everything we trained on like rectal* [artesunate]*, drugs so that we can use them because if we don’t practise we can easily forget how to use them.”* (CHW, Uganda)

*“You might find that at first these people were using gloves …* [then] *they say, ‘aah aah after all now [I] am an expert I can do a RDT without gloves.’ So you find that they just forget the issue of gloves and use bare hands.”* (Health facility staff, Uganda)

### Support supervision

In Uganda and Zambia, districts supported health centre staff, whilst at health facility level, supervisors were tasked with strengthening CHWs’ skills in case management, drug storage, reporting and promoting community engagement. In South Sudan, where the County Health Department and health facilities did not have the capacity to carry out consistent supervision, Malaria Consortium programme offices provided support to CHWs and their supervisors. In Uganda, “parish coordinators” were introduced as supplementary supervisors to provide support to CHWs on a more regular (usually monthly) basis and to assist with the compilation and submission of CHW data. Parish coordinators were CHWs who were selected by their peers in the same catchment area to fulfil this role. In all countries supervision occurred through quarterly meetings at health facilities and supervisor visits to CHWs’ homes.

The importance of timely support supervision immediately following training to ensure correct practices in the community were demonstrated by multiple respondents. Quarterly meetings at local level and/or at health facilities were identified as key to support supervision; an opportunity for CHWs to share experiences and solutions, build their confidence, raise concerns and refresh their skills. Home visits, especially the first visits conducted within two months after the initial training, were described as an indication of appreciation and particularly motivational for CHWs, and associated with marked improvement in sustaining of CHWs’ performance. The visits were also seen as a mechanism for strengthening links between CHWs and health facilities. Community recognition from being visited by supervisors was cited as a source of pride for CHWs.

*“Our supervisors supervise us very well we can’t even complain wherever we face any problem they sit us all down and ask about our challenges.”* (CHW, Zambia)

*“… I think those [home visits] were also very positive because we were able to go down to the community and see how they are basically doing their work. This motivated the VHTs a lot … They would see a car parked there and they would say, ‘the VHT has been visited,’ so it was a big thing.”* (Malaria Consortium staff, Uganda)

In areas where there was weaker support supervision, CHWs described feeling discouraged and demoralised.

*“Supervision is not consistent and when they don’t come to see what we are doing we become demoralized and sometimes when they don’t come we feel that what we are doing is not very important.”* (CHW, Uganda)

*“… some of the communities are just hostile. Instead of appreciating, they are just abusing the VHT saying after all you were given drugs to come and treat us. These people *[the CHWs]* get demoralised.”* **(**District health official, Uganda)

The most commonly mentioned barriers to effective support supervision included: availability of funds/transport allowances, difficulty in accessing CHWs due to poor roads or hard to reach locations, especially in the rainy season and time required, availability of health facility staff. In South Sudan insecurity and flooding were highlighted as additional challenges which affected supervisors’ ability to support CHWs. Weak supervisors were less frequently mentioned as a hindrance to strong supervision, which a few respondents attributed to insufficient training on supervisory skills during the initial training.

*“Unfortunately the health workers had a very big load and lack of logistics so they were not able to do it. It was later when the parish coordinators were trained and they came on board that actually supervision started.”* (Health facility staff, Uganda)

“Buddying up” weak and strong supervisors and introducing “peer CHWs” (high performing CHWs who could support those who are struggling) were recommendations on how to strengthen support supervision and improve quality of care. In South Sudan, a few CHWs described the latter approach as a mechanism that evolved during implementation when supervisors were not available.

*“When CDDs do not perform well, we call other CDDs to help in assisting in treatment …”* [All– *Agree*] (CHW, South Sudan)

### Data management

CHWs in all countries were trained to complete monthly registers detailing patient age, sex, respiratory rate, RDT result (Uganda and Zambia), diarrhoea, treatment given, referral and outcome. The register is submitted to supervisors, collated at health facility level and reported upwards and in some cases, the data included into the national Health Information Management System (HMIS). Generally, the quality of the data according to the majority of respondents was considered to be of an acceptable standard (ranging from “50% accurate” to “excellent”), which had also improved over time.

*“I can say it is good quality because the CHWs are trained and are supervised time and again to make sure the data is complete, correct and consistent.”* (District health official, Zambia)

In Uganda and Zambia, inaccuracies in the CHW registers were reportedly due to poor numeracy skills, lack of sufficient training on the tool, CHWs forgetting how to correctly enter the data and human error which was suggested to largely relate to fatigue and, busyness with other activities. Commodity stock outs were cited as the main reason for not completing registers for CHWs in Uganda and Zambia. In South Sudan, low literacy was identified as affecting data quality; in response later versions of the CHW register were redesigned to be pictorial to reduce errors. When gaps were identified in the data, supervisors explained that they would consult with CHWs for clarification. The integration of community level data within the HMIS remains a priority in South Sudan and Zambia.

*“Of course we are human and it is very possible for us to make mistakes. There are times when we attend to clients at night and having come from deep sleep, you record wrong details. All these are corrected until they correspond to what really happened.”* (CHW, Zambia)

*“… when we run out of drugs, we also stop writing reports.”* (CHW, Zambia)

Challenges cited in relation to data submission frequently referred to insufficient funds/transport availability and distance to health facilities, which for remotely located CHWs was exacerbated during the rainy season. Where supervisors were unavailable, CHWs discussed more reluctance to submit their data. In South Sudan, insecurity affecting Unity state led to extreme measures to collect data and provide CHWs with commodities.

*“Transport is another issue which is making our work very difficult because we are subjected to walking long distances … for 12 kilometres. Even my shoes are worn out.”* (CHW, Zambia)

*“Collecting data on time was affected by inaccessibility and insecurity. One time, one of the counties w[as] cut off …* [the road] *was mined and it took time to de–mine it.”* (Malaria Consortium staff, Unity state)

Data usage reportedly varied between and within countries. More similarities were identified in Uganda and Zambia where the data was commonly used to identify the general community case load against predicted numbers; to plan outreach activities; and to monitor CHW performance by comparing cases with diagnosis and treatment data. This use demonstrated the value placed on CHW data by health facilities and district health teams. In South Sudan, responses indicated that health facilities lacked the capacity to utilize data.

*“Sometimes, when there is an outbreak of a disease in the community, it’s very easy to bring such information to the health centre and indeed it's my prime responsibility as a VHT to communicate such information and it’s also our work to ensure that we fight such an outbreak in our community.”* (CHW, Uganda)

*“We use it for analysing disease incidence among villagers or zones. So, we see where we have the heaviest disease burden or areas that need urgent relief … that helps us to plan malaria activities very much so that we can try to mitigate. Whenever reports indicate an outbreak, we also intensify our activities so that people are aware of malaria messages and prevention.”* (Health facility staff, Zambia)

In all countries there was an apparent need to strengthen the competencies of health professionals to improve data analysis and usage whilst also emphasizing value to encourage incorporation of the data into planning activities.

“*...data collection skills and analysis should be improved through capacity building trainings for the iCCM staff* [at health facilities] *and partners.”* (Health facility staff, South Sudan)

*“When we realised that data was not forthcoming we got* [health facility] *staff to collect the data so when they saw them they realised the importance of this data for planning purposes.”* (Malaria Consortium staff)

### Commodities and supply chain

The aim of integrating commodities into the public sector supply chain conflicted in practice with the need to distribute a comprehensive package of commodities quickly to lower levels of the health system in Uganda and Zambia during the initial stages of implementation. In South Sudan, it was necessary to deliver commodities directly to CHWs due to the absence of an effective national supply chain. Periods of stock outs were widely reported across countries and were more acute during the rainy season. Community level respondents agreed that stock outs negatively impacted community perceptions of iCCM and even CHWs, who in some areas bore the brunt of the community`s frustrations, which several CHWs linked to reduced motivation and consequent attrition. Health facility staff observed a significant impact on their workload, which they reported increased when caregivers could not obtain treatment within their communities. Recommendations to strengthen the supply chain discussed basing quantification on consumption data and increasing buffer stock, especially in the rainy season. In Zambia, health facility staff across districts commented that there is an interchange of MOH commodities and iCCM supplies. For instance, if CHWs experience stock outs of anti–malarial drugs, the health centre may ‘top up’ their supplies; equally if health centres run low on RDTs or anti–malarial drugs, they may use iCCM stock.

Drug acceptability over time appeared to be universally high, with beneficiaries commenting on the quality and effectiveness of the medication.

*“When the child gets sick … and if found with malaria, they give this child a dose and if you follow their instructions, the illness will be treated and that’s why we like this medication.”* (Beneficiary, Uganda)

Community acceptance of RDTs was cautious initially, increasing over time (RDTs were only included as part of iCCM in Zambia and the Western region of Uganda). In Uganda and Zambia this was attributed to a few concerns over the purpose of taking blood, specifically that the sample would be used for HIV testing or “satanic” purposes. CHWs and health facility staff appreciated the introduction of RDTs in identifying malaria cases prior to providing treatment and reducing the amount of anti–malarial drugs administered presumptively.

*“… the results are instant. So, children can start treatment immediately. This has helped us as the children do not become too sick as was the case in the past.”* (Beneficiary, Zambia)

*“...the introduction of iCCM helped us to treat what we are sure of. The use of the RDTs has helped to identify fever and … has boosted our morale and self–esteem plus confidence before the caregivers … Here Coartem is not wasted; it’s only given to the proved RDT positive cases.”* **(**Health facility staff, Uganda)

### Community involvement and support

It was widely reported that beneficiaries embraced iCCM and welcomed the introduction of the programme, which brought “relief” to beneficiaries, especially in remote and hard to reach locations. Most commonly this appreciation was attributed to the reduction in long distances for seeking care, followed by the availability of free treatment accessible at any time of day. Timely treatment, not having to “dress up” or face long wait times or stock outs at clinics were other appreciated aspects.

“The communities welcomed the programme and the selection of CHWs in that it made accessibility to health care possible … some children used to die because most parents were failing to take them to the clinic due to long distances involved.” (CHW, Zambia)

*“When a child falls sick at night, we are able to rush to a VHT for quick treatment without being bothered by lack of money and distance as it used to be in the past.”* (Beneficiary, Uganda)

Despite what some beneficiaries considered a short training period, generally trust in CHWs increased over time as beneficiaries observed the CHWs’ effective case management and quality of care.

*“The initial response from the community was negative because these were normal community members, how do they start treating our children? … but the VHTs sensitised the community about the medicine they have and later community accepted them especially when community saw that their drugs can heal their children.”* (Community leader, Uganda)

Across countries utilisation was reportedly high, with respondents in South Sudan commenting on a shift from traditional medicine to accessing CHW services in their communities. In Zambia several CHWs remarked that uptake had increased as awareness of iCCM amongst caregivers grew. Health facility staff in Uganda and Zambia reported that the number of malaria, pneumonia and diarrhoea cases (most commonly malaria) reduced significantly following the introduction of iCCM in their catchment areas. Community level respondents gave anecdotal accounts of a reduction in child deaths.

*“Community Health Workers are doing a good job and this also has reduced the number of deaths in children under the age of five because long before iCCM was introduced children under the age of five used to die a lot due to long distances to health centres but ever since the programme was introduced, the numbers of deaths have reduced.”* (Beneficiary, Zambia)

The majority of respondents across the three countries felt that most beneficiaries heeded CHWs’ referrals to a health facility, often due to concerns over the health complications if the child were not treated. According to community level respondents, caregivers faced a range of barriers to accessing health facilities following a CHW’s referral, including: distance, cost (transport, consultation and treatment); which were the most frequently cited by respondents residing in remote locations. Long waiting times and stock outs at health facilities were considered other challenges, as were tribal differences and language barriers. Consequently, caregivers would alternatively access private pharmacies nearby or traditional healers if unable to travel to a health facility.

CHW workload mostly varied between “manageable” and “heavy” with a few reports that CHWs encountered challenges in combining CHW service provision with personal duties. Community leadership support for CHWs was identified as influencing overall community support for CHWs, which was manifested most commonly as service utilisation, verbal thanks, followed by recognition, small tokens of appreciation and in Zambia, assistance with cultivating plots of land. In Uganda, a common misconception amongst beneficiaries was the belief that CHWs received a salary, which CHWs explained discouraged caregivers from providing support. CHW attrition was reportedly due to an absence of financial incentives (often referred to in the data as “motivation”) and weak community support. A frequent recommendation was to enhance sensitisation on the role communities can play in supporting iCCM.

*“Only areas where headmen are serious about this programme … are areas where the headmen are very committed and they make sure that the community helps you.”* (CHW, Zambia)

*“… attrition among CHWs does happen in our community because some of them feel that they are not receiving enough support from the community; especially during farming season. They are failing to provide for their families since they spend much of their time providing health care services to the community at the expense of their families.”* (Community leader, Zambia)

### Behaviour change communication

The role of community leaders in delivering behaviour change communication (BCC) messages was cited as key across countries. Where implemented, community dialogues, which involved providing a platform for beneficiaries to discuss aspects of iCCM, were viewed as successful.

*“I think the most effective communication method was through the LC* [community leader] *because he is near the community members.”* (Health facility staff, Uganda)

“[The BCC strategy] *has helped in reducing cases of malaria in our communities. Initially, we also did a lot of mobilisation and community programmes, which helped inform people on the purpose and process of the iCCM programme. It has mostly been by community engagement.”* (Community leader, Zambia)

Community level respondents reported the value of BCC activities, specifically in promoting the use of CHWs, encouraging trust in western medicine and boosting CHW motivation. In Zambia and particularly in South Sudan, respondents across target groups observed that BCC had been instrumental in shifting attitudes towards western medicine and ultimately health seeking behaviour amongst caregivers. Several respondents also associated BCC effectiveness with increased service utilisation and in encouraging trust between caregivers and CHWs, who were viewed as successful in implementing change within their communities.

*“The regular meetings have really helped because the people have so much faith and trust in the CHWs now.”* (Community leader, Zambia)

*“… our being announced over the radio is an official introduction to the bigger world beyond our communities and this gives us recognition and morale.”* (CHW, Uganda)

*“Few people were now using traditional herbs for the treatment of children sickness in the villages.”* (Community leader, South Sudan)

Recurrent recommendations were to enhance BCC activities in terms of range and frequency, particularly in the early stages of implementation. Community structures, such as the Village Health Clubs in Uganda as well as community and religious leaders across countries were highlighted as optimal channels to disseminate BCC messages and engage communities.

*“...sensitisation should be widespread in the community and there should be genuine consultation.”* (Community leader, Zambia)

### iCCM integration

**Figure 1:**
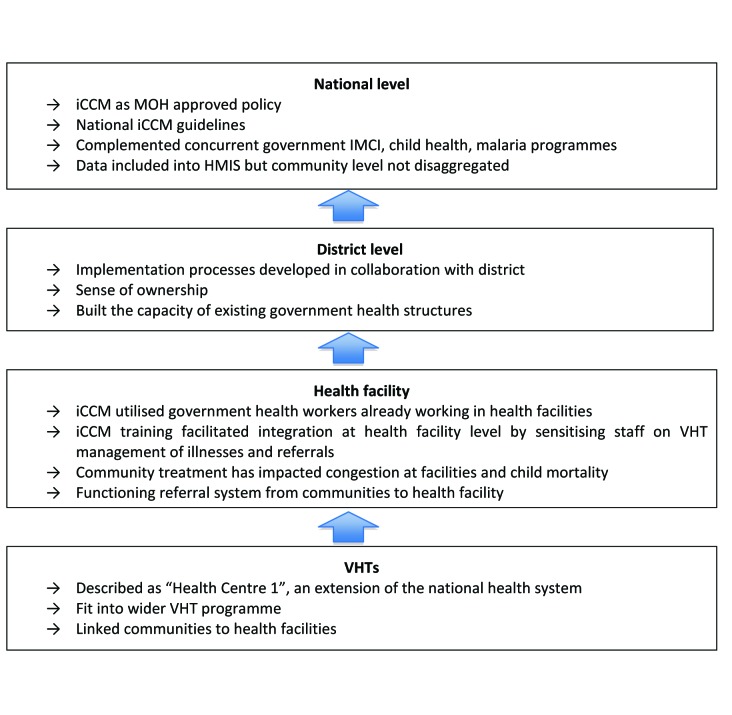
Multi–level iCCM integration as described by respondents in Uganda.

The consensus amongst respondents was that iCCM had been well integrated into the health system through implementation within existing health structures and in line with the MOH’s objectives to reduce under five morbidity and mortality. The collaboration between Malaria Consortium and local health teams promoted the transference of skills and implementation processes to the districts, building local capacity and according to several respondents in Uganda, creating a sense of ownership (see **Figure 1** for multi–level iCCM integration as described by respondents in Uganda).

Support from health facility staffs at all levels was reported to be generally high. In South Sudan, one recommendation was to strengthen the capacity of health workers and promote the procurement and distribution of second line treatment for severe cases of malaria, pneumonia, diarrhoea and malnutrition. Discussions around sustainability highlighted challenges such as commodities procurement, supporting the supply chain and motivating CHWs. Respondents at each level recognised that in order for iCCM to become sustainable, communities would need to support CHWs whilst the MOH at local, district, provincial and national levels would need to advocate for continued support of the programme, allocating funding and providing logistical support.

*“iCCM is a novel approach because previously we just used the IMCI. But iCCM encompasses the IMCI principles and also other principles to be able to manage the children. Further, I think this one also involves the use of the iCCM community members or volunteers and it goes a step further by empowering them with medication and skills to be able to treat or manage children at the community level.”* (MOH official, Zambia)

*“iCCM is using health systems that were already in place since its introduction. At community level, they are using the VHT system which started in 2004. So iCCM is well integrated in the Ugandan health care system … What is needed now is taking up the use of iCCM drugs in health facilities. Further, we exchange data with iCCM and this is a sign of integration”* (District health official, Uganda)

## DISCUSSION

Respondent experiences overall indicated that implementation followed national guidelines or agreed approach. Deviations from the implementation plans were often in response to operational challenges. New processes such as the introduction of parish coordinators to address gaps in CHW support supervision (in Uganda) and how data are utilised by health facilities and lower level MOH to inform community outreach activities which occurred during implementation (though not specifically described in official guidelines) could be formally incorporated into the iCCM country programmes. Generally, the data from Uganda and Zambia have more commonalities, most likely due to their socioeconomic similarities, compared to South Sudan, which has recently emerged from a protracted conflict.

The overall findings are discussed here around the themes of access, quality, demand, impact and policy, which are based on a results framework adapted by the CCM Operations Research group to assess intermediate results during the outcome of evaluation phase of iCCM implementation^2^.

### Access

As described throughout the data, respondents widely appreciated that health services had been brought to communities, facilitating timely and accessible treatment which they anecdotally reported reduced under–five cases and deaths and lessened the caseload at health facilities. Community level respondents located in remote and hard to reach locations far from health facilities (>5 km) who faced significant barriers in accessing health services, including distance and transportation costs, were even more vocal in their praise of iCCM services, indicating that future implementation should continue to target these communities. However, the success of iCCM in reducing under–five morbidity and mortality in hard to reach communities partly relies on the capacity of health facilities serving these catchment areas to appropriately manage referred cases. The factor that most affected the availability of services was stock outs. Enabling a regular supply, which is quantified based on actual consumption, especially in relation to seasonal increases in disease incidence, should remain a priority.

### Quality

Selecting appropriate CHWs through a democratic, participatory selection process, adopting participatory training approaches to promote sound comprehension of the course content, and providing refresher training and regular support supervision appeared to result in stronger CHW service provision and motivation which has been found by others^16^. This in turn encouraged caregivers to access treatment, and empowered CHWs to effectively deliver community health services to a high quality standard, as well as to improve the quality of the data they submitted. The motivational effect of supervision was identified in a previous study which found that, *“Many participants considered supervision to be the most important factor for maintaining a functional cadre of motivated CHWs stressing its potential for conveying a sense of belonging and connectedness to the program”* [10].

While there is a need to strengthen a few areas of the programme at community level, particularly CHW use of respiratory timers and the completeness and accuracy of the data amongst weaker CHWs, respondents agreed that the quality of care and specific commodities was generally high. Support supervision was highlighted as an area both in the data and other literature^3^ that could be enhanced with regards to frequency and an emphasis on CHW home visits. This would also require stronger support from sub–national levels of the health system, specifically more prioritisation and logistical support, in the form of transport, and funding.

### Demand

There was a high level of acceptability of iCCM and CHWs by communities and health facilities, which led to a high demand for services. This appeared to be closely related to the extent and quality of sensitisation and engagement during the initial phase of implementation. At health facility and district levels, this took the form of collaboration during the planning stages. For health facility staff, acceptability and support seemed to hinge very much on being adequately informed about the programme and financial facilitation for their participation throughout. At the community level, where members selected CHWs through a transparent, participatory process, and were sufficiently sensitised on the CHW role and services, utilisation and demand for services was reportedly higher. The data highlights how iCCM addressed community needs, which in turn affected demand for these services. Initially, although iCCM was generally embraced by communities, there were some concerns around the short duration of the CHW training that community members felt may have been insufficient. Nevertheless, these doubts diminished over time as CHWs proved themselves capable of managing cases. It was noted that demand was negatively impacted by stock outs, which in some instances altered community perceptions of iCCM, leading to a loss of faith in the programme among community members and decreased morale as identified by a few CHWs. Substantial anecdotal evidence as presented in the Results section indicates an increase in CHWs as the first point of contact for sick children following the introduction of iCCM, which is supported by the findings of the endline surveys from all three countries. The praise and appreciation for iCCM voiced by respondents across communities and numerous requests for the programme to continue further indicate that iCCM services will continue to be in high demand in communities where malaria, pneumonia and diarrhoea remain major threats to the lives of children.

Ownership of the programme contributed to the successful implementation of the programme, community demand for services and has implications for sustainability. Specifically, when communities feel that the CHWs they selected are “theirs”, caregivers were more likely to access and support the programme. When district/county health teams fully own the programme and view activities such as support supervision as their responsibility (rather than a parallel activity conducted by NGO partners), iCCM will be better integrated into the health system and ultimately more sustainable. A sense of ownership at central level (MOH, MCH, NMCP, national drug authorities) can encourage officials to allocate funding for iCCM, whilst at sub–national level officials may be more likely to provide logistical support and prioritise resources to sustain iCCM. Ownership and use of iCCM data at central level would promote incorporation into the HMIS tool, while at lower levels, it could encourage motivation and prioritisation by demonstrating the value of the data at national level. Moreover, this could inspire the sub national level to support consistent data collection as well as data analysis and use, for instance for drug quantification, assessing the performance of CHWs, filling support supervision gaps and planning community health outreach activities.

### Impact

The positive impact of iCCM reverberated throughout the data from anecdotal accounts of reductions in child morbidity and mortality from both community and health facility level respondents; across the board, respondents discussed how iCCM had made a significant difference to community child health in their districts. Health facility staff expressed appreciation over how outpatient departments were less congested, with fewer cases (of the three target diseases) presenting at their facilities. BCC messages on seeking timely and appropriate treatment reportedly led caregivers to favour CHWs over traditional healers as first point of contact and to follow CHW referrals to a health facility according to respondents in all three countries.

### Policy

The MOHs across countries already recognise iCCM as a priority in the fight against malaria and other fatal childhood illnesses, but support may need to be maintained for a significant allocation of the budget for iCCM commodities and activities, which may inspire the districts to follow suit to sustain the implementation of this lifesaving programme.

The use of mobile phone technology, usually referred to as mHealth, is currently being explored in many countries as a means of facilitating and strengthening core aspects of iCCM including data management, supervision and stock management. It is recognised that this approach has the potential to address many of the commonly encountered bottlenecks to effective implementation of iCCM programmes.

Sustainability was an implicit theme throughout discussions with respondents, with specific reference to procurement, the supply chain, support supervision and CHW motivation (financial and otherwise) and support. If the aim of an iCCM programme is to be integrated into the existing health structure as far as possible, funding and support from the national and sub national level MOH is vital. Through all levels of the health system, recognising iCCM as an important strategy for childrens’ health, through sensitisation, regular planning and a clear understanding of roles and responsibilities is key to supporting the programme in the long term. Challenges such as human resource gaps at health facilities, particularly in rural areas, hinder effective support supervision and effective management of referred cases, which in turn affects VHT morale and motivation highlight the need to support peripheral health facilities as an integral part of creating a sustainable and well accepted iCCM programme. The distribution of supplies is another aspect that could be strengthened through collaboration with the public supply chain and the districts to promote timely delivery of supplies to avoid stock outs and maintain strong community support for the programme.

### Recommendations

Experiences of iCCM implementation sourced from the 646 individuals included in this multi–country participatory evaluation provide insight into how iCCM can be strengthened from community to national level. Key programmatic recommendations to enhance the effectiveness, quality and sustainability of future iCCM implementation and scale up for the MOH and implementing partners are presented in **Table 3**. Many of the recommendations that emerged from this study are consistent with other iCCM programme evaluations [16–19].

**Table 3:** Specific recommendations for future iCCM implementation

Theme	Recommendations
**Central level preparation**	• Clear timeframe for the development/ revision of guidelines with multiple stakeholders
	• Effective collaboration with the MOH at district level in the detailed planning for implementation start up
	• Contingency funds for unforeseen costs such as health promotion and disease prevention training prior to iCCM training for CHWs who have not received it
**District level introduction**	• Close collaboration with districts and central level implementers from outset in terms of planning, costing and implementation
	• Sensitise all health facility staff (where possible)
**CHW selection**	• Timely and enhanced sensitisation prior to CHW selection to promote familiarity with the guidelines, transparency on the voluntary nature of the role and community participation.
	• Involve districts, health facility staff in monitoring selection process
	• CHW selection criteria to include an age range (ie, 18–45)
**Training and capacity building**	• Maintain a participatory and interactive approach to training, utilise videos, visits to health facilities where possible. Translate key terms into local languages
	• Adapt training materials to the context and participants’ level of comprehension/literacy and numeracy levels
	• Allocate more time during the training to focus on challenging areas, specifically pneumonia diagnosis and the use of respiratory timers, data management and stock management and for trainers, enhancing supervisory skills
	• More focus on the newborn care component where this is part of the national policy
	• Extend the CHW training from six to ten days to enable better digest of content and practice in application, particularly relating to challenging parts of the course
	• Conduct a standardised test at the end of the training and provide a certificate for those who have passed
	• Provide refresher training for CHWs which focuses on problem areas identified through supervision
**Support supervision**	• Supervisors to visit CHWs (home visits) within one month of initial training to review application of new skills/knowledge in practice and to motivate CHWs
	• Regular support supervision at frequent intervals (quarterly)
	• Prioritise support supervision within the MOH so that logistical support is provided and sustained
	• Promote district ownership and logistical support for supervision activities as far as possible, including integrating with other activities such as data collection/management
	• Link support supervision to CHW register data to identify gaps in knowledge, stock and assess CHW performance
	• Move towards competency based supervision and tools • Introduce supervisors for CHW supervisors– ie, another level of supervision
**Data management**	• Sensitise CHWs, health facility staff and DHMTs on the importance and uses of iCCM data, for instance in quantifying stock, identifying missing data in CHW registers, assessing CHW performance, planning disease control/community health activities
	• Build the data analysis and management capacity of health facility staff and DHTs
	• Clarify and communicate roles and responsibilities among health facility staff to support better prioritisation of data management activities
	• Advocate for, and support the process for, the integration of community level iCCM data into the HMIS tool
	• Provide equipment to CHWs to facilitate data submission (eg, bicycles, gumboots, rain coats)
	• Document data submission systems that have worked and share with implementers
	• Encourage health facilities using data as feedback for mapping trends and quantifying stock, to share their experiences with facilities that do not do this
	• Create mechanisms and templates for districts to feedback relevant iCCM data summaries to health facilities and CHWs
	• Scale up mHealth for data management and as a means of supervision and motivation of CHWs in locations where mobile phone networks have sufficient reliability and capacity
	• Provide CHWs with solar panels where possible to establish a consistent power supply to charge phones
**Commodities and supply chain**	• Integrate iCCM commodities into national public supply chain from outset
	• Support for improved commodity flow through the district, with an emphasis on integration with the district supply chain, where this can be properly supported
	• Adjust quantity of RDTs, artemisinin–based combination therapy and amoxicillin based on actual consumption data and continue to revise in line with data generated to avoid stock outs
	• Supply health facilities with buffer stock, especially during the rainy season (RDTs, artemisinin–based combination therapy)
	• Share distribution records with the district as needed to facilitate ownership of the process
**Community involvement and support**	• Emphasise sensitisation and regular community consultation through community leaders and community health committees or similar; specifically on the role of CHWs, the scope of iCCM services and the role of the community in supporting CHWs
	• Encourage a more sustainable mechanism through which communities can support CHWs, for instance cultivating land, assisting with chores, or material contributions– positive examples could be shared with other communities
**Behaviour change communication (BCC)**	• Roll out BCC activities prior to iCCM implementation in communities to raise awareness about CHWs, iCCM services to promote demand
	• Contextualise BCC activities as much as possible based on existing information and initiatives; special consideration for hard to reach areas so that the most effective methods for those locations are utilised (ie, community gatherings vs radio if coverage is poor)
	• Utilise interactive approaches such as community dialogues, storytelling/posters
	• Emphasise specific key messages during BCC activities: ▫ Promote timely and effective utilisation of iCCM services ▫ The importance of community participation in the CHW selection process ▫ The voluntary nature of the CHW role ▫ The role of the community in supporting CHWs ▫ Clarification on the purpose of RDTs, specifically what the blood is being tested for to avoid misconceptions
**Management and coordination**	• Enhance information sharing of results and surveys between implementing partners and DHTs
	• Document roles and responsibilities ie, through Memorandums of Understanding (MoUs) to serve as a record of agreed processes
	• Improve collaboration and more frequent communication between implementing partners, the district and the different levels of the health system to enable effective implementation and address challenges
**Integration**	• Advocate for the MOH and donors to prioritise iCCM in terms of funding and logistical support
	• Facilitate visits from central MOH and donors both at start–up and during implementation to share experiences and promote the value of the programme
	• Prioritise addressing the gaps in support supervision and data management
	• Strengthen the supply chain to facilitate the timely and frequent delivery of iCCM commodities
	• More collaboration with the district during planning stage on how best to integrate iCCM activities into district level plans and budget
**Technical and geographical scope**	• Strengthen current iCCM activities before widening the scope in terms of age or coverage
	• Continue to/ prioritise hard to reach locations
	• Gather and share more evidence to inform appropriate CHW/ population ratios
**Evaluation**	• Evaluate programme impact and feedback findings to all levels of the MOH, partners and communities
	• Involve the districts closely in all monitoring and evaluation activities
	• Strengthen local capacity to undertake monitoring and evaluation activities

CHW – community health worker, iCCM – integrated community case management, MOH – Ministry of Health, DH(M)T – district health (management) team, HMIS – health information management system, RDT – rapid diagnostic test

## CONCLUSIONS

This qualitative study offers a valuable contribution in understanding the “hows” of implementation, and uncovers many implications for improved feasibility and acceptability of iCCM in practice. The findings clearly demonstrate that community support to iCCM and CHWs is necessary for sustained health benefits while keeping a focus on strengthening and “enabling” the public health system. The use of participatory methodologies enabled the scope of the research enquiry to be context specific and to be inclusive of stakeholders at all levels.
